# Uncovering Key Genes Associated with the Short-Winged Trait in Faba Bean (*Vicia faba* L.) Through Re-Sequencing and Genome-Wide Association Studies (GWASs)

**DOI:** 10.3390/ijms26062733

**Published:** 2025-03-18

**Authors:** Haitian Yu, Chaoqin Hu, Xin Yang, Qiong Li, Yubao Wang, Zhengming Dai, Jie Cun, Aiqing Zheng, Yanhua Jiang, Qinfang Wang, Meiyuan Lv, Feng Yang, Yuhua He

**Affiliations:** 1 Institute of Food Crops, Yunnan Academy of Agricultural Science, Kunming 650205, Chinayangfeng@yaas.org.cn (F.Y.); 2 Department of Agricultural, Food and Nutritional Science, University of Alberta, Edmonton, AB T6G 2P5, Canada; 3 Qujing Academy of Agricultural Sciences, Qujing 655000, China

**Keywords:** *Vicia faba*, short-wing trait, low outcrossing rate, genotyping by sequencing, genome-wide association study (GWAS), SNP loci

## Abstract

Faba bean (*Vicia faba* L.) is a globally significant legume valued for its applications in food, vegetables, and green manure, yet its high outcrossing rate (30–80%) poses challenges for production development. A rare short-winged trait identified in Yunnan, China, offers promise for developing low-outcrossing varieties, reducing outcrossing rates to below 5%. Scanning electron microscopy (SEM) and transmission electron microscopy (TEM) analyses revealed that the epidermal cells of normal wing petals are conical, while those of short-wing petals are tubular. This study examined 200 F_2_ lines from crosses between ‘K0692’ (short-winged) and ‘Yundou 1183’, as well as ‘Yundoulvxin 1’ (short-winged) and ‘Yundou 1183’. The GWASs identified 10 SNP loci across chromosomes 2, 3, 4, and 5, with SNP_chr4::1013887633 explaining 22.20% of the wing trait variation. Key candidate genes were identified, such as VFH_III145120, which influences floral identity; and VFH_III149200, associated with epidermal differentiation. GO enrichment analysis demonstrated significant gene involvement in RNA localization, ribosome biogenesis, and preribosome metabolism, while KEGG analysis linked these genes to pathways in amino acid, nucleotide, and purine metabolism; ubiquitin-mediated proteolysis; and protein processing in the endoplasmic reticulum. These findings lay a foundation for breeding low-outcrossing faba bean varieties and enhancing sustainable faba bean cultivation.

## 1. Introduction

Faba bean (*Vicia faba* L.) is a globally significant legume known for its high nutritional value and environmental benefits. It serves multiple purposes, including human food, livestock feed, and green manure, which enhances soil fertility through nitrogen fixation [1]. This dual functionality aligns with sustainable agricultural practices, supporting crop rotation systems and reducing reliance on synthetic fertilizers. The adaptability of faba bean to diverse climatic conditions further underscores its importance in improving food security, particularly in regions vulnerable to climate change [1,2].

Despite these advantages, faba bean cultivation faces challenges, particularly due to high outcrossing rates ranging from 7% to 82% [3]. This outcrossing can introduce unwanted genetic variability, complicating breeding programs aimed at developing stable, high-yielding varieties and increasing susceptibility to diseases and pests. Thus, managing outcrossing rates is essential for enhancing the agronomic traits of faba bean.

**Figure 1:**
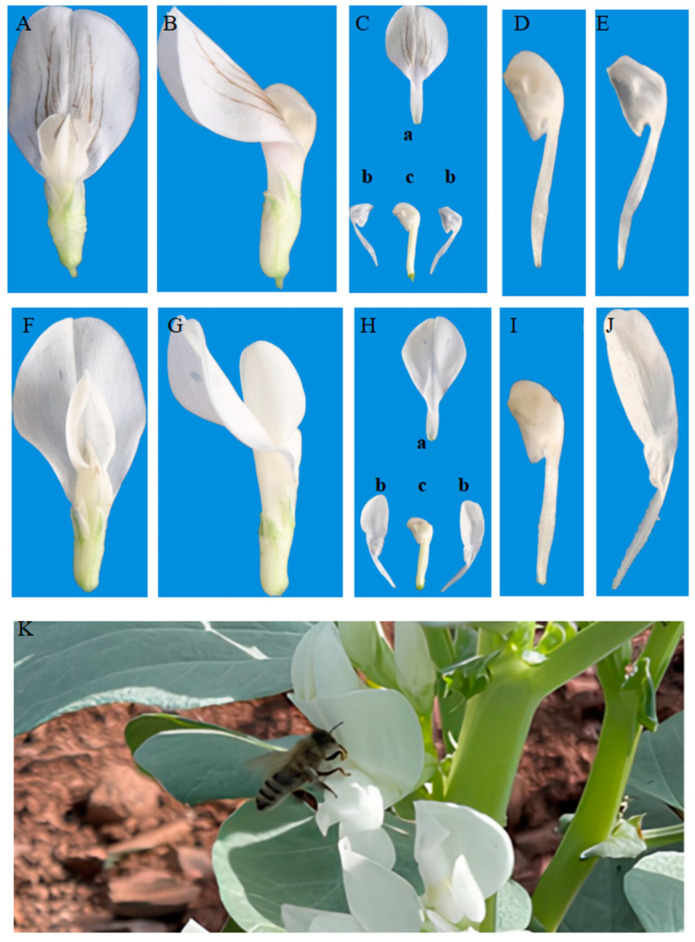
Comparison of short-winged flowers (**A**–**E**) and regular flowers (**F**–**J**): (**A**,**F**) top view of the corolla; (**B**,**G**) side view of the corolla; (**C**,**H**) diagram of the corolla (standard petal (a), wing petal (b), and keel petal (c)); (**D**,**I**) keel petal; (**E**,**J**) wing petal; and (**K**) one of the honeybee’s nectar-gathering behaviors on faba bean flowers (facilitating effective cross-pollination), where the bee uses the wing petals as a foothold, landing between the two wing petals to collect nectar.

Recent efforts have focused on identifying genetic resources to mitigate high outcrossing rates. A rare germplasm from Yunnan, China, exhibits a short-winged trait (Figure 1A–E) that significantly reduces its outcrossing rate to below 5% [4]. Normal-wing petals (Figure 1H(b)) are longer than the keel petal (Figure 1H(c)) and serve as a landing platform for honeybee foraging activity [5] (Figure 1K). In contrast, the short-winged petal (Figure 1C(b)) is identical in shape and length to the keel petal (Figure 1C(c)), eliminating a stable landing site for honeybees and restricting their ability to access nectar [4,6]. Pollinators, particularly honeybees, typically extract nectar from the junction of the standard petal and the wing petal, using the wing as support while foraging (Figure 1K), which is recognized as an effective mechanism for nectar transmission among faba bean plants [5]. However, the short-winged trait poses a disadvantage for insect-mediated foraging as bees cannot land on the short-winged petals, thereby reducing cross-pollination.

Based on nearly 40 years of breeding records, the short-winged faba bean has exhibited minimal variation in the field, making it an excellent germplasm for maintaining a low outcrossing rate and ensuring production stability [4,6]. While the high outcrossing rate in faba bean has been widely documented for its positive effects—such as increased heterozygosity, improved plant vigor, and enhanced yield [7]—it also presents challenges. The coexistence of allogamous and autogamous pollination complicates breeding strategies, as conventional methods used for either self-pollinated crops or highly outcrossing species like maize are not directly applicable to faba bean [8]. The short-winged trait offers a unique opportunity to develop low-outcrossing faba bean varieties through molecular breeding techniques, enabling the creation of more genetically stable germplasm and facilitating breeding strategies suitable for self-pollinated crop improvement in faba bean.

Floral morphology evolution plays a critical role in plant reproductive strategies and has been extensively studied in botany [9]. Among floral structures, petals significantly influence pollinator attraction and reproductive success [10]. Petal morphology can vary widely among species, adapting to specific ecological niches and pollination mechanisms [11]. Furthermore, the cellular structure of petals impacts their overall functionality; the arrangement, size, and shape of epidermal cells can affect light reflection, water retention, and the flower’s ability to attract pollinators [12,13]. Previous studies have shown significant differences in epidermal structures among flower types, underscoring the importance of cellular architecture in floral design [14]. Bailes et al. (2018) employed transmission electron microscopy (TEM) and scanning electron microscopy (SEM) techniques to investigate the phenotypic diversity of epidermal cells in faba bean petals. Their findings suggest that conical (papillate) epidermal cells provide better anchoring for pollinating insects, thereby enhancing their residence time on such petals [15].

Genetic research has emerged as a rapid and effective approach for molecular breeding [16]. Studies on other leguminous crops, such as the early investigation in 1923, indicated that the wing phenotype in pea is controlled by a single gene, designated “k” [17], and the mapping of this gene has been reported [18]. In *Lotus japonicus*, the KEW1/kew1 gene regulates petal wing formation and influences epidermal cell development [19]. This gene is expressed in both wing and standard petals, with expression levels in wing petals being significantly higher, particularly during late floral development [20].

Currently, there is a lack of reports on short-winged faba bean germplasms and the regulation of short-winged petals in this species. The large nuclear genome of faba bean, approximately 13 Gb, has contributed to delays in whole-genome sequencing and high-throughput genetic studies compared to other major crops [21]. Recent advancements, such as the comprehensive genomic information reported by Jayacodi et al. (2023), provide a valuable reference for molecular genetic studies, including the construction of genetic maps using hybrid populations [22].

To further investigate morphological differences, this study employed SEM and TEM to examine the epidermal cells of short-winged and normal-winged varieties. A total of 200 F_2_ lines were derived from crosses between short-winged and normal-winged varieties. Utilizing advanced re-sequencing techniques and genome-wide association studies (GWASs), several single nucleotide polymorphism (SNP) loci were mapped across chromosomes. This research not only identified these SNP loci, but also provided a comprehensive analysis of candidate genes associated with them, revealing a complex genetic landscape related to wing morphology. These findings underscore the significance of the identified SNPs in regulating wing morphology and their potential utility in marker-assisted selection for breeding programs.

## 2. Results

### 2.1. Analysis of Segregation of the Wing Trait in F_2_ Populations

The segregation ratios for the wing trait in the two F_2_ populations were 1389:483 for normal-winged to short-winged in the ‘Yundoulvxin 1’ (short-winged) × ‘Yundou 1183’ cross, as well as 1624:531 for the ‘K0692’ (short-winged) × ‘Yundou 1183’ cross (Table 1). The observed ratios of the normal-winged to short-winged individuals were 2.88:1, 3.06:1, and 2.97:1 for ‘Yundoulvxin 1’ × ‘Yundou 1183’, ‘K0692’ × ‘Yundou 1183’, and the combined populations, respectively, as shown in Table 2. These ratios conform to the expected segregation ratio of 3:1.

### 2.2. Microscopic and Genetic Basis of the Wing Trait

#### 2.2.1. Scanning Electron Microscopy (SEM) and Transmission Electron Microscopy (TEM) of Petal Epidermal Cells

**Figure 2:**
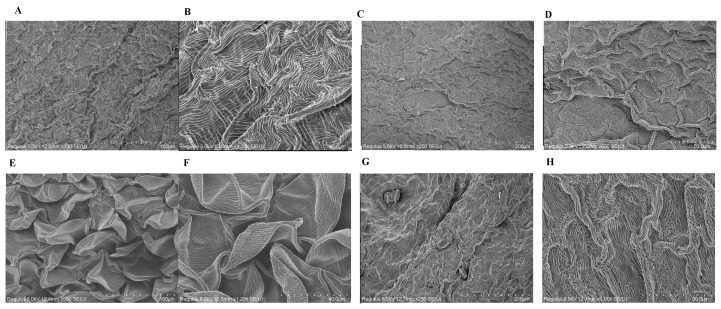
Scanning electron microscope results of the epidermal cells: the wing petal (**A**,**B**,**E**,**F**) and keel petal (**C**,**D**,**G**,**H**) epidermal cells of the short-winged faba bean (**A**–**D**) and normal-winged faba bean (**E**–**H**). (**E**,**F**) Conical cells; others, tabular cells.

**Figure 3:**
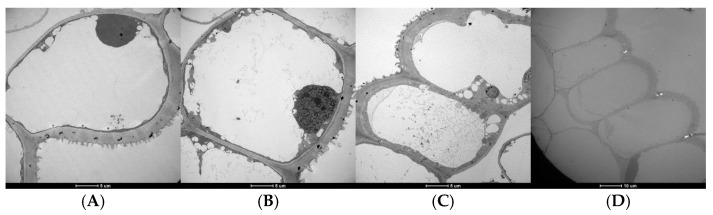
Transmission electron microscope scan results of the epidermal cells: wing petal epidermal cells of the short-winged faba bean (**A**,**B**) and normal-winged faba bean (**C**,**D**). (**A**,**B**) Tabular cells; (**C**,**D**) conical cells.

SEM analysis revealed that the epidermal cells of the wing petal in the short-wing variant were tabular (Figure 2A,B), whereas the normal-wing variant featured conical cells (Figure 2E,F). These observations were further confirmed by TEM, which showed tabular cells in the short-wing variant (Figure 3A,B) and conical cells in the normal-wing variant (Figure 3C,D). In contrast, the epidermal cells of the keel petal were consistent across both variants, exhibiting tabular cell morphology (Figure 2C,D,G,H).

#### 2.2.2. Genome-Wide Association Study on the Wing Trait

##### Population Characteristic Analysis and LD Analysis

To identify the genes associated with the short-winged trait, we sequenced 200 individuals from the F_2_ populations, comprising 50 short-winged and 150 normal-winged individuals, which were consistent with a 3:1 ratio. A total of 1072.16 G reads were generated, with 96.94% of these reads achieving Q30 quality or higher and a GC content of 41.31%.

**Figure 4:**
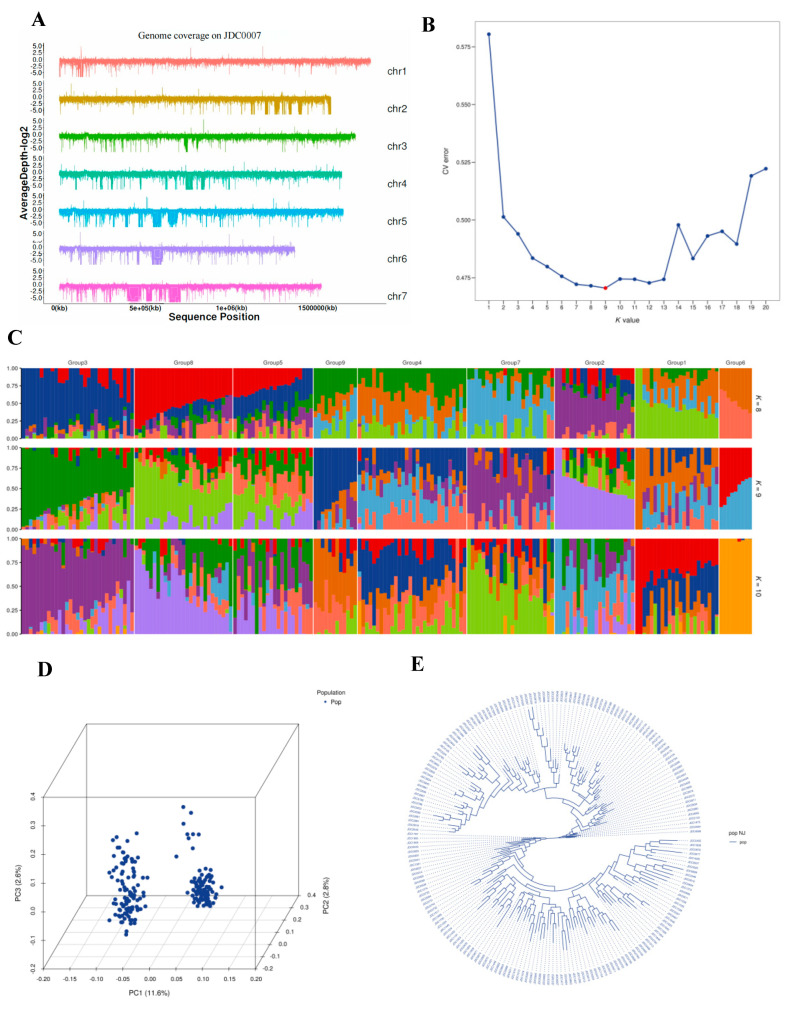
The genome structure and genotyping analyses of 200 F_2_ individual populations. (**A**) Genome structure; (**B**) cross-validation diagram of the SNP dataset; (**C**) population structure analyzed by STRUCTURE at K = 8, 9, and 10; (**D**) principal component analysis of the population; and (**E**) phylogenetic tree of the population. The red point in (**B**) indicates the structural simulation results, showing that k = 9 minimized the cross-validation (CV) error.

Using the Hedin2 genome v1 [22] as the reference, we identified 32,339,886 SNPs and 1,126,648 InDels. After applying filtering criteria (missing data < 30% and minor allele frequency [MAF] > 5%), a total of 488,121 SNPs and 14,931 InDels remained for analysis. The highest density of SNPs was observed on chromosome 5, while the lowest density of SNPs was detected on chromosome 7, with an average marker density of 41 SNPs per Mb (Figure 4A).

To explore the population structure and genotype characteristics of the 200 tested F_2_ individuals, we employed ADMIXTURE software Version 1.3.0. This analysis utilized an unsupervised maximum likelihood estimation model and involved cross-validation to determine the optimal number of subpopulations (k = 1–20). The structural simulation results indicated that k = 9 minimized the cross-validation (CV) error (Figure 4B,C). Consequently, we selected a k value of 9 to assess the genetic structure of the 200 F_2_ individuals. In the principal component analysis (PCA) of these individuals, the first two principal components explained 14.4% of the total genetic variation (Figure 4D).

Significant genetic variation was observed among the 200 F_2_ individuals in this study. To further investigate the genetic differentiation among genotypes, we performed clustering based on the neighbor joining (NJ) method. Consistent with the ADMIXTURE results, the stratified NJ tree revealed distinct subclusters, despite all of the individuals belonging to a larger overall group (Figure 4E). Nine subclusters were identified in the NJ tree, aligning with the nine major subgroups from the ADMIXTURE analysis (Figure 4B,C). The resulting Q matrix at k = 9 was used for subsequent marker-trait association mapping.

All of the high-quality SNP markers were employed to estimate the linkage disequilibrium (LD) within the F_2_ population. In our analysis, the maximum observed *r*^2^ value was 0.45, and the half-decay distance would, theoretically, correspond to the physical distance, at which *r*^2^ decayed to half of this value (0.225). However, even at the maximum calculated distance of 500 kb, the *r*^2^ did not decay to 0.225, indicating a relatively slow LD decay rate in our population. Therefore, we used 500 kb as the LD half-decay distance. For visualization and interpretation, we referred to an *r*^2^ threshold of 0.34, which reflects the approximate background level of LD across the genome under the given distance constraints (Supplementary Figure S1).

##### Genome-Wide Association Analysis

To identify the key genetic loci and candidate genes associated with the short-winged trait, we conducted genome-wide association studies (GWASs) using three models: GLM, MLM, and FarmCPU. The GLM was included as a baseline model without controlling for the population structure, while MLM accounted for both the population structure and kinship to reduce false positives. FarmCPU was selected for its ability to effectively control confounding while maintaining statistical power through using a multi-locus mixed model approach. The rMVP software (Version 1.0.6) package was used to analyze the associations between SNP markers and the short-winged trait in the F_2_ population, enabling detection of significant marker-trait associations. A GWAS threshold of −*log* (P) = 7 was used in declaring significant marker-trait associations (MTAs).

- GLM Model

**Figure 5:**
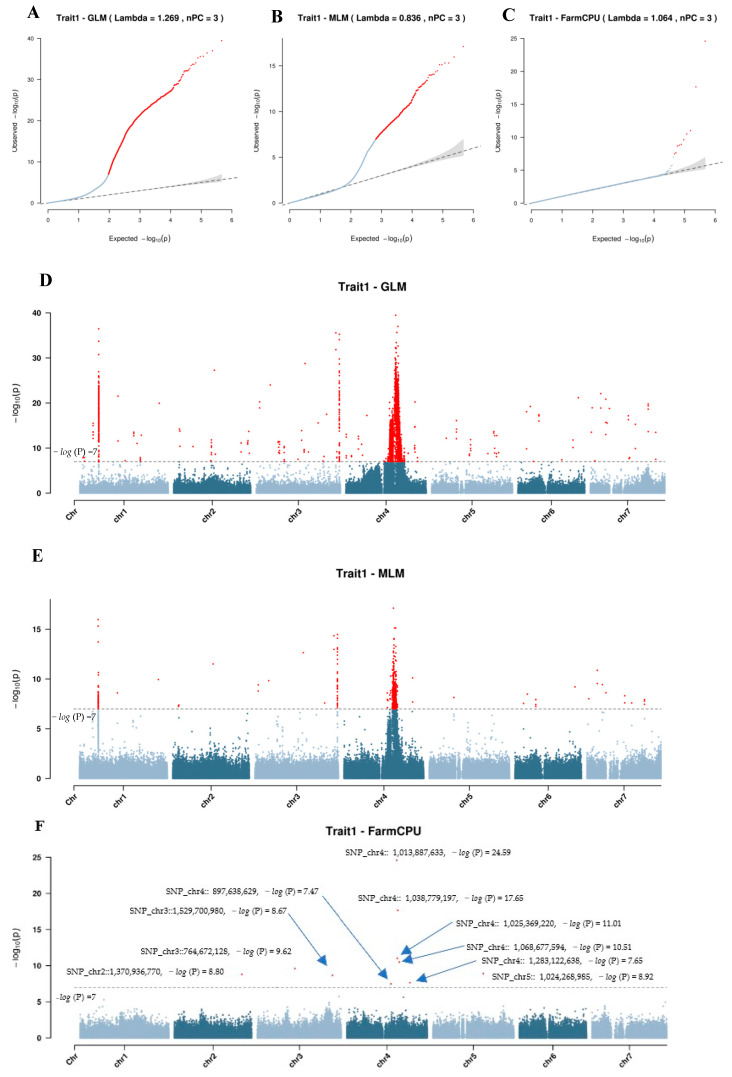
Loci related to the short-winged trait based on the GLM, MLM, and FarmCPU methods. (**A**,**D**) QQ and Manhattan plots of the GWAS results based on the GLM method; (**B**,**E**) QQ and Manhattan plots of the GWAS results based on the MLM method; and (**C**,**F**) QQ and Manhattan plots of the GWAS results based on the FarmCPU model. Red dots above the threshold line or deviating from the linear curve represent significantly associated loci.

Using the GLM model, a Lambda value of 1.269 was obtained with three principal components (nPC = 3) (Figure 5A). A total of 4958 SNPs across seven chromosomes and 45 InDels were identified. SNPs were distributed as follows: 361 on chromosome 1; 25 on chromosome 2; 118 on chromosome 3; 4390 on chromosome 4; 23 on chromosome 5; 15 on chromosome 6; and 26 on chromosome 7 (Figure 5D). Among these, 53 InDels and 2319 SNPs explained over 10% of the trait variation, while a subset—5 InDels, 105 SNPs on chromosome 1, 5 on chromosome 3, and 522 on chromosome 4—accounted for more than 20% of variation. Notably, 4 SNPs on chromosome 1 and 14 on chromosome 4 explained over 30% of variation, with the SNP at position SNP_chr4::1013887633 explaining the highest variation at 49.37%.

- MLM Model

With the MLM model, the Lambda value was 0.863 with three principal components (nPC = 3) (Figure 5B). A total of 716 SNPs and 11 InDels showed significant association with the wing trait in faba bean. The SNP distribution included 86 on chromosome 1; 5 on chromosome 2; 61 on chromosome 3; 542 on chromosome 4; 2 on chromosome 5; 9 on chromosome 6; and 11 on chromosome 7 (Figure 5E). A subset of 277 SNPs from chromosomes 1 and 4, and sca659 and sca1426, explained over 10% of trait variation. Key loci on chromosome 1 (SNP_chr1::386258298, SNP_chr1::386258299, SNP_chr1::386258476, and SNP_chr1::386330156) and chromosome 4 (SNP_chr4::1013887633, SNP_chr4::1038779197, and SNP_chr4::1060355967) explained over 20% of variation, with SNP_chr4::1013887633 reaching 26.85%.

- FarmCPU Model

Using the FarmCPU model, the Lambda value was 1.064 with three principal components (nPC = 3) (Figure 5C). Ten SNPs significantly associated with the short-winged trait were identified (Figure 5F, Table 2). One SNP on chromosome 2 (SNP_chr2::1370936770) explained 9.29% of variation. Two SNPs on chromosome 3 (SNP_chr3::764672128 and SNP_chr3::1529700980) explained 9.29% and 6.18% of variation, respectively. Six SNPs on chromosome 4, including SNP_chr4::1013887633, which accounted for 22.20% of variation, also exhibited significant associations. The final SNP, located on chromosome 5 (SNP_chr5::1024268985), explained 11.78% of the variation in the wing trait.

Significant associations were identified between the SNP pairs on chromosomes 2 and 4 (Supplementary Table S1 and Supplementary Figure S2, respectively), with distinct odds ratios and contributions to the variance (*R*^2^). A notable association was observed between SNPs chr4::1013887633 and chr4::1068677594, showing an odds ratio of 533.85 and a *p*-value of 0.0261, explaining 2.03% of the phenotypic variance. These associations suggest potential loci interactions that may play a role in influencing the trait of interest.

##### Candidate Gene Analysis

The Q-Q plot generated from the FarmCPU model demonstrated that most points aligned along the diagonal, and the genomic inflation factor (λ) for FarmCPU was 1.064, which is close to 1, suggesting an appropriate balance between false positives and false negatives. This indicates a robust model fit for identifying genetic associations with the wing trait in the F2 population. Therefore, FarmCPU was determined to be the best model for identifying significant SNPs and performing candidate gene blasting. Utilizing this model, significant SNPs were analyzed, and a 500 kb confidence interval on each side of these SNPs in the Hedin2 genome v1 was used to search for candidate genes linked to the short-wing trait. In total, 52 candidate genes were identified (Table 3), although none were previously annotated in faba bean. Functional annotations for these candidate genes were assigned based on the Universal Protein Database (UniProt), providing insights into their potential roles in trait expression.

On chromosome 2, three novel candidate genes were identified, with gene-VFH_I185240 associated with DNA integration, metal ion binding, and ribonuclease activity; gene-VFH_I185160 linked to the regulation of organ growth and protein ubiquitination (protein ubiquitination:regulation of seed growth:ubiquitin protein ligase activity); and gene-VFH_I185200 had an unknown function.

Chromosome 3 yielded ten novel candidate genes, including gene-VFH_II126600, which was associated with transcription factor activity and DNA binding near SNP_chr3::764672128. Nine additional genes were identified around SNP_chr3::1529700980, including gene-VFH_II242840 and gene-VFH_II242880 with RNA–DNA hybrid ribonuclease activity; gene-VFH_II242760 was involved in vesicle-mediated transport; and gene-VFH_II126600, gene-VFH_II242920, and gene-VFH_II242960 were related to transcription regulation and DNA binding.

On chromosome 4, thirty-two candidate genes were identified across multiple intervals. Two genes near SNP_chr4::897638629, gene-VFH_III128960, and gene-VFH_III129000 were of unknown function. Eight genes in the region of SNP_chr4::1013887633 were also uncharacterized. In contrast, SNP_chr4::1025369220 contained gene-VFH_III147240, which was associated with post-translational modification; gene-VFH_III147280, which was linked to nucleotide transport and metabolism; and the other six genes were uncharacterized. Additionally, SNP_chr4::1038779197 included gene-VFH_III149200, which was found to be involved in signal transduction mechanisms and protein kinase activity. Within the candidate intervals of SNP_chr4::1068677594, ten genes were identified. Among them, gene-VFH_III153600, gene-VFH_III153640, gene-VFH_III153560, and gene-VFH_III153520 were linked to translation, ribosomal structure, and biogenesis, while gene-VFH_III153400 was associated with post-translational modification, protein turnover, and chaperone activity. Gene-VFH_III153480 was found to be involved in signal transduction mechanisms. Four other genes in this region—gene-VFH_III153320, gene-VFH_III153280, gene-VFH_III153440, and gene-VFH_III153360—have unknown functions. Additionally, three genes within the interval of SNP_chr4::1283122638—gene-VFH_III185920, gene-VFH_III185880, and gene-VFH_III185960 also lacked functional annotations.

Chromosome 5 analysis revealed four candidate genes within the interval of SNP_chr5::1024268985, with all of them having unknown functions in faba bean. However, gene-VFH_IV148120 and gene-VFH_IV148160 were predicted to encode subtilisin-like proteases in Arabidopsis thaliana, while gene-VFH_IV148200 likely encodes an inactive purple acid phosphatase. The function of gene-VFH_IV148240 remains unreported. These findings provide a basis for future exploration of the genetic basis of the short-wing trait and offer candidate genes that warrant further functional validation.

##### Gene Ontology (GO) Enrichment Analysis

**Figure 6:**
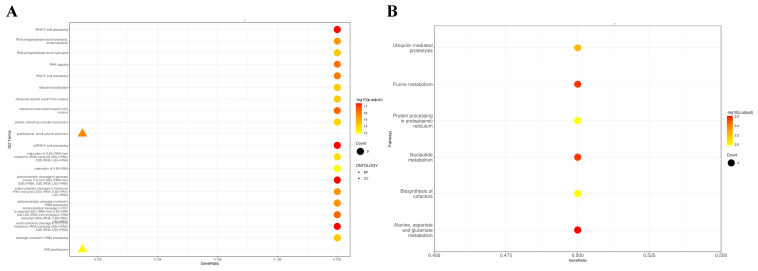
GO (**A**) and KEGG (**B**) analyses of the genes associated with short-winged traits via the FarmCPU model. (**A**) GO Enrichment Analysis, candidate genes were enriched in biological processes and molecular functions; (**B**) Kyoto Encyclopedia of Genes and Genomes (KEGG) Annotation of candidate genes significantly involved in metabolic pathways.

The Gene Ontology (GO) enrichment analysis of the candidate genes identified significant enrichment in two primary functional categories (along with the preribosome compound metabolic process (Figure 6A)): RNA/rRNA and protein localization activity. Within these categories, two terms related to cellular components were identified: 90S preribosome (GO:0030686) and preribosome, small subunit precursor (GO:0030688). The biological process category included 18 terms related to rRNA processing and protein localization. These terms are as follows: cleavage involved in rRNA processing (GO:0000469); endonucleolytic cleavage in 5′-ETS of tricistronic rRNA transcript (SSU-rRNA, 5.8S rRNA, LSU-rRNA) (GO:0000480); endonucleolytic cleavage in ITS1 to separate SSU-rRNA from 5.8S rRNA and LSU-rRNA from tricistronic rRNA transcript (SSU-rRNA, 5.8S rRNA, LSU-rRNA) (GO:0000447); endonucleolytic cleavage involved in rRNA processing (GO:0000478); endonucleolytic cleavage of tricistronic rRNA transcript (SSU-rRNA, 5.8S rRNA, LSU-rRNA) (GO:0000479); endonucleolytic cleavage to generate mature 5′-end of SSU-rRNA from (SSU-rRNA, 5.8S rRNA, LSU-rRNA) (GO:0000472); maturation of 5.8S rRNA (GO:0000460); maturation of 5.8S rRNA from tricistronic rRNA transcript (SSU-rRNA, 5.8S rRNA, LSU-rRNA) (GO:0000466); ncRNA 5′-end processing (GO:0034471); protein-containing complex localization (GO:0031503); ribosomal small subunit export from nucleus (GO:0000056); ribosomal subunit export from nucleus (GO:0000054); ribosome localization (GO:0033750); RNA 5′-end processing (GO:0000966); RNA capping (GO:0036260); RNA phosphodiester bond hydrolysis (GO:0090501); RNA phosphodiester bond hydrolysis, endonucleolytic (GO:0090502); and rRNA 5′-end processing (GO:0000967).

##### Kyoto Encyclopedia of Genes and Genomes (KEGG) Annotation

The Kyoto Encyclopedia of Genes and Genomes (KEGG) annotation indicated that the metabolic pathways of the candidate genes are primarily associated with key biochemical processes, specifically alanine, aspartate, and glutamate metabolism (ko00250); nucleotide metabolism (ko01232); purine metabolism (ko00230); ubiquitin-mediated proteolysis (ko04120); biosynthesis of cofactors (ko01240); and protein processing in the endoplasmic reticulum (ko04141) (Figure 6B).

## 3. Discussion

This study provides critical insights into the genetic basis of the short-winged trait in faba bean, highlighting its potential as a tool for reducing outcrossing rates and stabilizing crop performance. The segregation pattern observed in the F_2_ populations, with a 1:3 ratio of short-winged to normal-winged plants, suggests a single recessive gene controls the trait. This finding is consistent with previous studies on legumes such as *Pisum sativum* and *Lotus japonicus*, where similar short-wing traits are also governed by single recessive genes [17,19]. Given the 1:3 segregation ratio of short-winged to normal-winged plants in the F_2_ population, we selected 50 short-winged and 150 normal-winged individuals to reflect this natural inheritance pattern. While a more balanced case–control design could improve statistical power, our approach ensures adequate representation of the short-winged phenotype while maintaining the genetic diversity of the population. Future studies with larger sample sizes could further validate these findings and improve detection sensitivity.

Microscopic analyses using SEM and TEM revealed clear distinctions in epidermal cell morphology between the two wing types: short-winged variants showed tabular cells, while normal-winged variants exhibited conical cells. This morphological difference aligns with known floral adaptation mechanisms, as documented in *Lotus japonicus* studies, where the KEW1/kew1 gene influences wing petal morphology and cell development [19,20]. The consistency of tabular cell structure in keel petals across both wing variants suggests a conserved cellular architecture in these floral parts, possibly due to their functional roles.

The short-winged trait in the 200 selected F_2_ individuals, derived from two distinct crosses, was analyzed using a genome-wide association study (GWAS) approach. To account for potential population structure, we evaluated the selected individuals based on K values, which revealed nine distinct groups and is indicative of genetic variation within the population. This phylogenetic analysis supported the identified structure, aiding in the reduction in false positives in our GWAS results. Furthermore, our Q-Q plot analysis when using the FarmCPU model demonstrated a strong diagonal alignment of most data points, reinforcing the model’s effectiveness in addressing population structure [23]. The selection of GLM, MLM, and FarmCPU was based on their distinct advantages: GLM provides a simple linear association test, MLM accounts for kinship to reduce false positives, and FarmCPU enhances statistical power by integrating both fixed and random effects. To determine the most suitable model, we evaluated the genomic inflation factor (λ), where a value close to 1 indicates an appropriate balance between false positives and false negatives. The λ values for GLM (1.269) and MLM (0.836) suggested potential inflation and overcorrection, respectively, whereas the λ value for FarmCPU (1.064) was closest to 1, indicating well-controlled population structure effects. This, along with the well-calibrated Q-Q plot and clear signal resolution in the Manhattan plot, supports the choice of FarmCPU as the most reliable model for our analysis.

Our GWAS analysis identified 10 SNP loci that were significantly associated with the short-wing trait, with SNP_chr4::1013887633 accounting for 22.20% of the trait variance. Among the 49 candidate genes identified, most lacked prior annotations, and none had been previously associated with wing morphology in faba bean. Notable genes include VFH_III145120, which contains a NAM-related C-terminal domain linked to floral organ identity and lateral organ separation in model legumes such as *Medicago truncatula*. Microarray and real-time quantitative PCR analyses revealed that mutations in NAM down-regulate the expression of floral homeotic genes in *M. truncatula* [24]. These findings underscore the essential role of *NAM* in floral identity. NAM homologs in *Petunia* and *Arabidopsis* also play essential roles in floral patterning and differentiation, influencing the balance between cell division and expansion in reproductive organs [25,26]. Given its conserved function, VFH_III145120 in *Vicia faba* may similarly influence wing petal morphology by regulating genes involved in cell differentiation and expansion. Additionally, VFH_III149200, an inactive receptor kinase gene, likely contributes to epidermal differentiation, which was supported by the SEM and TEM observations of conical versus tabular cells between wing variants, paralleling findings in *Pisum sativum* (pea) and *Arabidopsis thaliana* [27]. Another significant candidate, VFH_I185160 on chromosome 2, corresponds to the E3 ubiquitin-protein ligase DA2 OS, which has been linked to floral development in *Arabidopsis* [28,29]. RNA-seq analysis identified over 200 proteins are present in the E3s mutant but absent in the wild type, while more than 300 proteins exhibited higher abundance in the mutant [29]. These findings highlight the crucial role of E3s. Given its association with SNP_chr4::1013887633, which explains 22.20% of trait variance, this gene may regulate key growth factors involved in petal expansion and final morphology.

The associations identified between the SNP pairs on chromosomes 2 and 4 suggest significant genetic interactions influencing the trait under study. Notably, the pair chr4::1013887633 and chr4::1068677594 showed a substantial positive association (OR = 533.85, *p* = 0.0261). While genes in the chr4::1013887633 region remain uncharacterized, those within the chr4::1068677594 region were associated with translation, ribosomal structure, biogenesis, post-translational modification, protein turnover, and chaperone activity. These findings indicate that the loci on chromosomes 2 and 4 may interact to modulate the trait, with some variants potentially acting as protective factors and others as risk enhancers. While high odds ratios like 533.85 are rare, they suggest that specific allelic combinations may have a pronounced effect. This extreme value may also be due to small sample sizes in certain genotype combinations, leading to inflated estimates. Further validation with larger sample sizes may help ensure the robustness of these findings.

The collective evidence suggests a hierarchical regulatory network in which NAM transcription factors (VFH_III145120) function upstream to orchestrate floral identity by regulating genes involved in cell differentiation and expansion. This regulation likely integrates with receptor kinase signaling (VFH_III149200) to fine tune the epidermal cell fate and structural modifications in wing petals. Additionally, post-translational mechanisms mediated by E3 ubiquitin ligases (VFH_I185160) contribute to protein turnover, ensuring precise control over floral organ growth. The interactions between loci on chromosomes 2 and 4 suggest a coordinated genetic framework, where some variants may enhance trait stability, while others introduce variability, shaping the final wing petal morphology in *Vicia faba*. However, further validation studies are required to confirm these associations and elucidate the underlying biological mechanisms.

Enrichment analysis when using GO and KEGG revealed key biological processes and metabolic pathways tied to the candidate genes, including RNA/rRNA processing, ribosome assembly, and protein localization. These processes are integral to morphological and developmental regulation, suggesting that the candidate genes identified may influence cellular architecture and wing morphology. Specifically, ribosome biogenesis and preribosome complex pathways, which are crucial for cellular growth and reproduction, emerged as significant. Mutations in ribosomal proteins (RPs) and ribosome biogenesis factors (RBFs) are known to impact plant growth and flowering times [30], aligning with our findings on the genetic regulation of floral traits.

Purine metabolism and ubiquitin-mediated proteolysis pathways, identified through KEGG analysis, are central to floral and cell differentiation. For instance, purine metabolism is critical for floral bud development, as shown in *Citrullus lanatus* studies, where gene expression in this pathway varies between male-fertile and sterile buds [31]. The ubiquitin-mediated proteolysis pathway plays vital roles in plant responses to environmental and developmental cues, including flooding, salinity, vernalization, and the functionality of the shoot apical meristem [28]. It is also involved in regulating plant responses to environmental factors and floral senescence, indicating the importance of protein turnover in floral organ development and maintenance [29,32]. The E3 ubiquitin ligase family, notably F-box genes like UFO (UNUSUAL FLORAL ORGANS) in *Arabidopsis* and its ortholog FIM (FIMBRIATA) in *Antirrhinum*, further illustrates the role of ubiquitin in flowering regulation; mutations in these genes can lead to various floral defects, indicating their multifaceted involvement in floral patterning [33,34,35,36]. Additionally, endoplasmic reticulum (ER)-associated protein degradation mechanisms, which are crucial for proper protein folding, may influence floral abscission and development [37], suggesting potential impacts on the wing morphology of faba bean.

The enriched GO terms highlight processes involved in RNA regulation and protein localization that could be pivotal for cell shape and differentiation. The significant enrichment of pathways related to amino acid and nucleotide metabolism, protein degradation, and cofactor biosynthesis further underscores these candidate genes’ regulatory roles in plant adaptation and morphology.

## 4. Materials and Methods

### 4.1. Plant Materials and Phenotypic Data Collection

Two F_2_ populations were created from crosses between ‘K0692’ (short-winged) and ‘Yundou 1183’ (n = 2156), as well as between ‘Yundoulvxin 1’ (short-winged) and ‘Yundou 1183’ (n = 1872). The two populations were used for segregation analysis. For the genome-wide association study, 200 F_2_ individuals from both populations were selected for re-sequencing (Supplementary Table S2). The parental lines ‘K0692’ and ‘Yundou 1183’ were also utilized for scanning electron microscopy (SEM) and transmission electron microscopy (TEM) analyses.

The F_2_ populations and the parental lines were planted at the Field Station of the Yunnan Academy of Agricultural Sciences in 2021. Wing type phenotypes were recorded at the flowering stage. Young leaf samples from all of the F_2_ individuals were collected at flowering and stored at −20 °C for future analysis. Fully opened flowers from the parental lines ‘K0692’ and ‘Yundou 1183’ were collected for SEM and TEM studies.

### 4.2. Scanning Electron Microscopy (SEM) of the Epidermal Cells on Wing and Keel Petals

To analyze the petal epidermis, SEM was performed on the wing and keel petals of the parental lines ‘K0692’ and ‘Yundou 1183’, following the protocol established by Xiao et al. [38]. Fresh petals from fully blooming flowers were selected, and central sections (approximately 0.5 × 0.5 mm) were cut and immersed in a 2.5% (*v*/*v*) glutaraldehyde solution (Biosharp, Hefei, China) at 4 °C overnight. The samples were then washed three times with phosphate buffer solution (pH 7.2, (Sigma-Aldrich, St. Louis, MO, USA)) for 10 min each. Subsequently, the petal samples underwent dehydration in a graded ethanol series (30%, 50%, 70%, 90%, 95%, and 100%), with each step lasting 10 min. The samples were treated twice with isoamyl acetate for 15 min each. After dehydration, samples were dried using a critical point dryer (EM CPD 300; BAL-TEC Inc., Balzers, Liechtenstein), with liquid carbon dioxide being used as the transitional fluid. Finally, the samples were mounted on metal stubs, sputter coated with gold, and examined using a scanning electron microscope (Hitachi SU-8100, Tokyo, Japan).

### 4.3. Transmission Electron Microscopy (TEM) of the Epidermal Cells on Wing Petal

Transmission electron microscopy (TEM) was conducted on the wing petals from the parental lines ‘K0692’ (short-winged) and ‘Yundou 1183’, according to the protocol by Tajuddin et al. [39]. Samples were initially fixed in a 2.5% glutaraldehyde solution (Electron Microscopy Sciences, Hatfield, PA, USA) prepared in 0.1 M phosphate-buffered saline (PBS) (Sigma-Aldrich) at pH 7.0 for two hours under vacuum to improve penetration. Following primary fixation, samples were rinsed with PBS multiple times, then subjected to secondary fixation with 2% osmium tetroxide (Electron Microscopy Sciences) for lipid preservation. For dehydration, samples were passed through a graded ethanol series, with each step lasting 15 min, and they were then transitioned to propylene oxide (Electron Microscopy Sciences). The samples were subsequently infiltrated with resin while being continuously rotated to ensure uniform penetration. After infiltration, samples were polymerized in epoxy resin at 70 °C overnight. The hardened resin blocks were sectioned to a thickness of 70–90 nanometers using an ultramicrotome (EM UC7, LEICA, Wetzlar, Germany). Sections were stained with uranyl acetate (Sigma-Aldrich) and lead citrate (Electron Microscopy Sciences) before examination under a transmission electron microscope (TECNAL SPIRIT G2, FEI, Hillsboro, OR, USA).

### 4.4. Genomic DNA Library Preparation and Sequencing

Total genomic DNA was extracted from the 200 selected F_2_ individual plants using the DNeasy Plant Pro Kit (Qiagen, Hilden, Germany), following the Quick-Start protocol (Qiagen.com/HB-2522) (Qiagen). DNA quantity and quality were evaluated using a NanoDrop 2000 spectrophotometer (Thermo Fisher Scientific, Waltham, MA, USA) and a Quantus™ Fluorometer (Promega, Madison, WI, USA). Only DNA samples meeting quality standards (OD260/280 = 1.8–2.0, OD260/230 ≥ 2.0, and concentration ≥ 25 ng/µL) were selected for sequencing library construction. DNA integrity and contamination were assessed via 1% agarose gel electrophoresis.

For library preparation, 0.5 µg of DNA per sample was used. Libraries were prepared as paired-end sequencing libraries for 10 × genome coverage using the Illumina NovaSeq™ X Plus platform (Illumina, San Diego, CA, USA). Library construction followed the TruSeq Nano DNA HT Sample Prep Kit protocol (Illumina, San Diego, CA, USA). Briefly, genomic DNA was sonicated to ~350 bp fragments, end-polished, A-tailed, and ligated to full-length adapters for sequencing on the NovaSeq X Plus. PCR amplification was performed, and the products were purified using the AMPure XP system. Library size distribution was assessed with an Agilent 2100 Bioanalyzer, and libraries were quantified by real-time PCR (3 nM). Paired-end sequencing was performed on the Illumina NovaSeq X Plus system at Shanghai Majorbio Bio-pharm Technology Co., Ltd (Shanghai, China).

### 4.5. Variant Discovery

Low-quality raw reads (mean Phred score < 20), i.e., reads with adapter contamination, and reads with unrecognizable nucleotides (N bases > 10) were trimmed or discarded using Fastp [40]. Trimmed reads were then mapped to the Hedin2 reference genome v1 [22] using BWA-MEME [41] with default parameters. Following a modified GATK Best Practices workflow [42], alignment BAM files were sorted using Samtools [43], and PCR duplicates were marked with MarkDuplicates. After base quality recalibration, germline variants (SNPs and InDels) were called across all samples using the Haplotyper and Gvcftyper programs in the Sentieon genomics tools [44]. Variants were filtered according to standard hard filtering parameters from the GATK Best Practices pipeline.

All variants were annotated with SnpEff [45]. SNPs and InDels were categorized based on chromosomal positions (intergenic regions, exons, introns, splicing sites, untranslated regions, and 1-kb upstream and downstream regions) and their functional effects (missense, start codon gain or loss, stop codon gain or loss, and splicing mutations). To minimize false positives in the SNP and genotype calling, several filtering steps were applied: (i) SNPs with more than two alleles were removed; (ii) SNPs with a mean depth across samples < 4 were excluded; (iii) SNPs with a minor allele frequency < 0.05 were discarded; (iv) SNPs were retained only if genotyped in at least 70% of samples; and (v) SNPs were pruned for population structure analysis based on linkage disequilibrium using Plink [46].

### 4.6. Phylogenetic Analyses

Maximum likelihood (ML) and neighbor joining (NJ) phylogenetic trees were constructed using IQ-TREE2 Version 2.1.2 [47] and FastTree Version 2.1.11 [48], respectively. The NJ tree was generated in FastTree with the GTR + Gamma model and 1000 bootstrap replicates, based on pruned SNP sites. ML analysis was conducted in *IQ-*TREE2 using the GTR + I + G4 model with 1000 bootstrap replicates.

### 4.7. Population Structure Analysis

Population structure was assessed using the unsupervised maximum-likelihood clustering algorithm in ADMIXTURE Version 1.3.0 [49] for k = 1 to k = 20 ancestral clusters with default parameters. Pruned SNPs were used for increased clustering accuracy.

### 4.8. Principal Component Analysis (PCA)

To visualize the genetic relationships among the 200 F_2_ lines, principal component analysis (PCA) was performed based on pruned SNPs using Plink Version 1.90b6.20 [46].

### 4.9. Population Diversity Indexes

Population divergence indexes were calculated using the populations program of Stacks Version 2.66.

### 4.10. Linkage Disequilibrium (LD) Analysis

To assess the LD decay across the genome, the squared correlation coefficient (*r*^2^) between loci pairs was calculated using PopLDdecay Version 3.42 [50], based on re-sequencing data. LD was estimated with a sliding window size of 50 kb, and a maximum inter-marker distance of 500 kb was set to calculate LD between SNP pairs. To minimize the bias caused by tightly linked loci, SNP pairs with *r*^2^ values greater than 0.5 were excluded from the analysis. The average *r*^2^ values were computed for pairwise SNPs within 500 kb bins, and these were then averaged across the genome to generate the LD decay curve.

### 4.11. Relatedness Analysis

The pairwise relatedness (kinship) between individuals was estimated using Plink Version 1.90b6.20 [51], based on SNP data.

### 4.12. Genome-Wide Association Analysis (GWAS)

Genome-wide association studies (GWASs) were performed to identify the loci associated with the short-winged trait using the R package ‘rMVP’ and by applying GLM, MLM, and FarmCPU models [52]. The first three principal components and a kinship matrix were incorporated as covariates in the models. Significance thresholds for *p*-values were set using a Bonferroni correction (0.05/n, where n is the total number of SNPs) to control the genome-wide type I error rate. Epistasis among significant loci was analyzed using Plink Version 1.90b6.20. To control multiple testing in the epistasis analysis, a false discovery rate (FDR) correction was applied to maintain a stringent genome-wide significance level. A GWAS threshold of − *log* (P) = 7 was used in declaring significant marker-trait associations (MTAs), which approximately corresponds to the Bonferroni-adjusted significance level given the total number of SNPs used in the analysis. This stringent threshold helps reduce false positives while ensuring robust association signals.

For loci identified as significant, a 500 kb flanking region on each side of the loci was extracted as the candidate region associated with the trait. Genes overlapping with these candidate regions were selected for further functional enrichment analysis.

## 5. Conclusions

This study identified the loci and candidate genes linked to the short-winged trait in faba bean, underscoring the genetic basis for this trait and its potential in reducing outcrossing rates. By integrating morphological observations with GWAS findings, this work provides a foundation for the molecular breeding of low-outcrossing faba bean varieties, promoting stable and reliable crop production. These insights contribute to the broader field of faba bean breeding and highlight the potential for developing sustainable, adaptable varieties under diverse environmental conditions.

## Figures and Tables

**Table 1:** Segregation of the wing traits in the F2 populations.

Population	Normal-Winged (N)	Short-Winged (S)	Total	N:S Ratio ^a^	Exp. N ^b^	Exp. S ^c^	Chi-Square ^d^	*p*-Value ^e^
‘Yundoulvxin 1’ (short-winged) × ‘Yundou 1183’	1389	483	1872	3N:1S	1404	468	0.64	0.42
‘K0692’ (short-winged) × ‘Yundou 1183’	1624	531	2155	3N:1S	1616.25	538.75	0.15	0.70
Total	3013	1014	4027	3N:1S	3020.25	1006.75	0.07	0.79

^a^ Expected ratio of the normal-winged to short-winged individuals in the F_2_ populations. ^b^ Expected number of the normal-winged individuals. ^c^ Expected number of the short-winged individuals. ^d^ Chi-square results comparing the observed and expected segregation. ^e^ A *p*-value greater than 0.05 indicates no significant difference.

**Table 2:** Loci related to the short-winged trait based on the FarmCPU method.

Chromosome	Position	Reference ^a^	Alternation ^b^	SE ^c^	*p*-Value ^d^	PVE ^e^
chr2	1,370,936,770	T	C	0.0190	1.58 × 10^−9^	0
chr3	764,672,128	T	A	0.0203	2.40 × 10^−10^	9.3%
chr3	1,529,700,980	T	G	0.0365	2.15 × 10^−9^	6.2%
chr4	897,638,629	G	A	0.0297	3.35 × 10^−8^	5.2%
chr4	1,013,887,633	C	T	0.0163	2.57 × 10^−25^	22.2%
chr4	1,025,369,220	A	G	0.0324	9.77 × 10^−12^	0
chr4	1,038,779,197	C	T	0.0195	2.22 × 10^−18^	17.0%
chr4	1,068,677,594	A	C	0.0101	3.11 × 10^−11^	7.4%
chr4	1,283,122,638	C	A	0.0206	2.25 × 10^−8^	0
chr5	1,024,268,985	A	G	0.0896	1.20 × 10^−9^	11.8%

^a^ Nucleotide of the reference genome. ^b^ Nucleotide mutations in the studied populations. ^c^ Standard errors for the percentage of variation explained (PVE) by each SNP. ^d^ Significance of the loci associated with phenotypic traits, where *p*-values less than 0.05 indicate significance. ^e^ Percentage of the phenotypic variation explained by the corresponding loci.

**Table 3:** Candidate gene list.

Gene ID	Chromosome	Strand	Start	End	Annotation
gene-VFH_I185160	chr2	+	1,370,573,643	1,370,577,612	E3 ubiquitin-protein ligase DA2 OS
gene-VFH_I185200	chr2	+	1,370,577,952	1,370,578,250	--
gene-VFH_I185240	chr2	+	1,370,586,133	1,370,595,083	--
gene-VFH_II126600	chr3	+	764,359,337	764,360,410	Ethylene-responsive transcription factor CRF3 OS
gene-VFH_II242640	chr3	+	1,529,265,502	1,529,266,471	--
gene-VFH_II242680	chr3	+	1,529,957,969	1,529,968,053	Sterol 3-beta-glucosyltransferase UGT80A2 OS
gene-VFH_II242720	chr3	+	1,530,074,691	1,530,075,183	--
gene-VFH_II242760	chr3	+	1,530,076,062	1,530,077,346	Vesicle-associated membrane protein 722 OS
gene-VFH_II242760	chr3	+	1,530,076,062	1,530,077,346	Vesicle-associated membrane protein 721 OS
gene-VFH_II242800	chr3	+	1,530,088,252	1,530,088,560	--
gene-VFH_II242840	chr3	+	1,530,088,586	1,530,088,906	--
gene-VFH_II242880	chr3	+	1,530,089,943	1,530,090,287	Uncharacterized mitochondrial protein AtMg00310 OS
gene-VFH_II242920	chr3	+	1,530,152,989	1,530,153,702	Zinc-finger homeodomain protein 2 OS
gene-VFH_II242960	chr3	+	1,530,183,734	1,530,184,447	Zinc-finger homeodomain protein 2 OS
gene-VFH_III128960	chr4	+	897,872,521	897,872,763	--
gene-VFH_III129000	chr4	+	897,910,440	897,912,825	--
gene-VFH_III144960	chr4	+	1,013,856,789	1,013,857,154	--
gene-VFH_III145000	chr4	+	1,013,857,243	1,013,861,427	--
gene-VFH_III145040	chr4	+	1,014,183,855	1,014,184,466	--
gene-VFH_III145080	chr4	+	1,014,260,687	1,014,262,444	--
gene-VFH_III145120	chr4	+	1,014,277,201	1,014,278,223	Glutathione S-transferase T3 OS
gene-VFH_III145120	chr4	+	1,014,277,201	1,014,277,560	--
gene-VFH_III145160	chr4	+	1,014,279,008	1,014,279,562	--
gene-VFH_III145200	chr4	+	1,014,289,183	1,014,290,059	Late embryogenesis abundant protein D-34 OS
gene-VFH_III147000	chr4	+	1,024,934,626	1,024,935,012	--
gene-VFH_III147040	chr4	+	1,025,114,272	1,025,114,726	--
gene-VFH_III147080	chr4	+	1,025,414,493	1,025,417,411	Vacuole membrane protein KMS1 OS
gene-VFH_III147120	chr4	+	1,025,427,305	1,025,427,901	--
gene-VFH_III147160	chr4	+	1,025,428,128	1,025,429,181	Putative ribonuclease H protein At1g65750 OS
gene-VFH_III147200	chr4	+	1,025,429,480	1,025,430,643	Glutathione S-transferase T3 OS
gene-VFH_III147240	chr4	+	1,025,451,849	1,025,452,856	Cullin-1 OS
gene-VFH_III147280	chr4	+	1,025,778,128	1,025,778,745	Adenylosuccinate lyase OS
gene-VFH_III149160	chr4	+	1,039,110,258	1,039,110,459	--
gene-VFH_III149200	chr4	+	1,039,111,995	1,039,112,210	Probable inactive receptor kinase At1g48480 OS
gene-VFH_III153280	chr4	+	1,068,451,765	1,068,460,306	Protein DWD HYPERSENSITIVE TO UV-B 1 OS
gene-VFH_III153320	chr4	+	1,068,460,834	1,068,461,079	--
gene-VFH_III153360	chr4	+	1,068,461,099	1,068,461,438	Snakin-1 OS = Solanum tuberosum OX
gene-VFH_III153400	chr4	+	1,068,485,623	1,068,487,425	Thioredoxin domain-containing protein 9 homolog OS
gene-VFH_III153440	chr4	+	1,068,772,610	1,068,775,302	Sister chromatid cohesion protein SCC2 OS
gene-VFH_III153480	chr4	+	1,068,789,249	1,068,792,353	Serine/threonine-protein kinase EDR1 OS
gene-VFH_III153520	chr4	+	1,068,929,535	1,068,931,137	Pumilio homolog 12 OS
gene-VFH_III153560	chr4	+	1,068,967,422	1,068,969,025	Pumilio homolog 9 OS
gene-VFH_III153560	chr4	+	1,068,967,422	1,068,969,025	Pumilio homolog 12 OS
gene-VFH_III153600	chr4	+	1,069,082,272	1,069,083,842	Pumilio homolog 12 OS
gene-VFH_III153640	chr4	+	1,069,110,264	1,069,111,871	Pumilio homolog 12 OS
gene-VFH_III185880	chr4	+	1,283,109,906	1,283,110,265	--
gene-VFH_III185920	chr4	+	1,283,164,266	1,283,168,067	Protein TIC 40, chloroplastic OS
gene-VFH_III185960	chr4	+	1,283,221,585	1,283,223,452	Rust resistance kinase Lr10 OS
gene-VFH_IV148120	chr5	+	1,024,025,212	1,024,028,413	Subtilisin-like protease SBT5.4 OS
gene-VFH_IV148160	chr5	+	1,024,573,401	1,024,592,701	Subtilisin-like protease SBT5.4 OS
gene-VFH_IV148160	chr5	+	1,024,573,401	1,024,580,420	Subtilisin-like protease SBT5.4 OS
gene-VFH_IV148200	chr5	+	1,024,680,906	1,024,685,005	Probable inactive purple acid phosphatase 27 OS
gene-VFH_IV148240	chr5	+	1,024,689,766	1,024,690,207	--

The positive and negative directions of the chain are indicated by the plus sign + and the minus sign −, respectively.

## Data Availability

Raw data were generated at the Institute of Food Crops, Yunnan Academy of Agricultural Science. Derived data supporting the findings of this study are available from the corresponding author, H.Y., on request.

## Supplementary Materials

The following supporting information can be downloaded at: https://www.mdpi.com/article/10.3390/ijms26062733/s1.
