# Supplementary material for: Uncovering Key Genes Associated with the Short-Winged Trait in Faba Bean (Vicia faba L.) Through Re-Sequencing and Genome-Wide Association Studies (GWASs)
Source: Int J Mol Sci. 2025 Mar 18;26(6):2733. doi: 10.3390/ijms26062733 (PMC11942482; doi:10.3390/ijms26062733)
Supplement: Supplementary file 1 [file ijms-26-02733-s001.zip › ijms-3508149-supplementary.pdf]

Supplementary Material

1 Supplementary Figures and Tables

1.1 Supplementary Figures

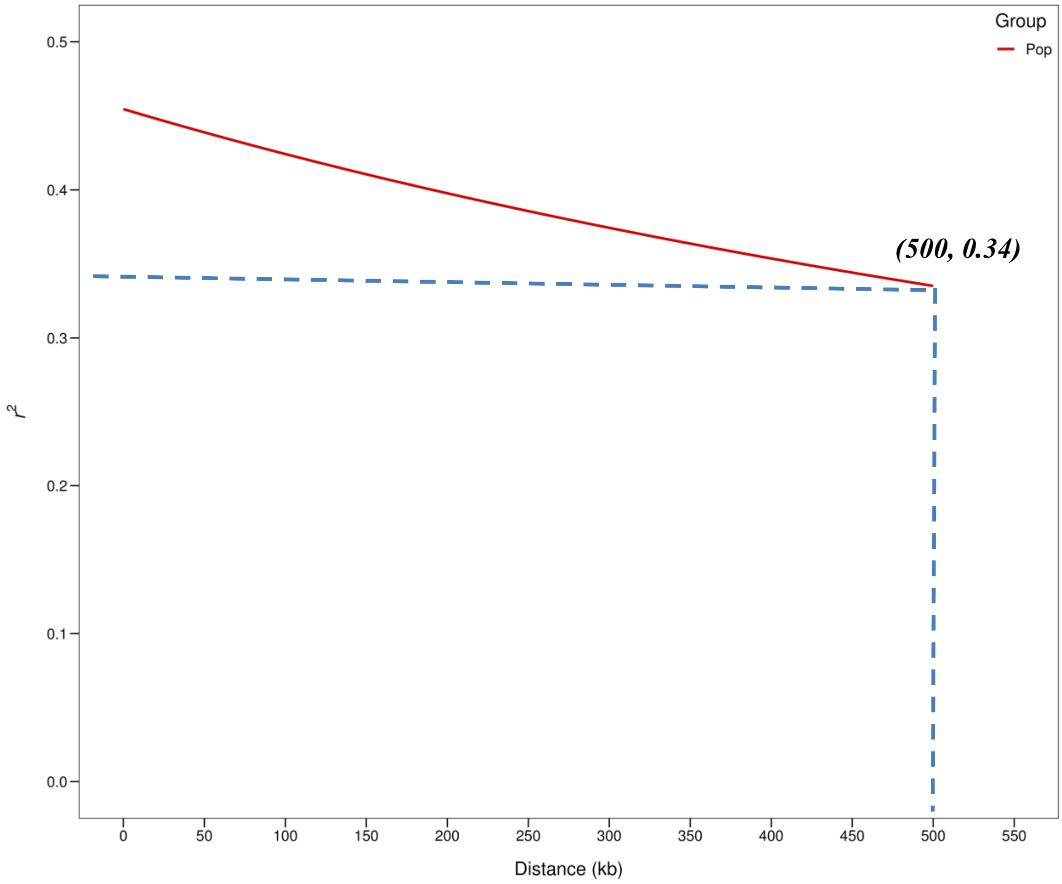

Supplementary Figure S1. Linkage disequilibrium (LD) of the tested F_2 populations.

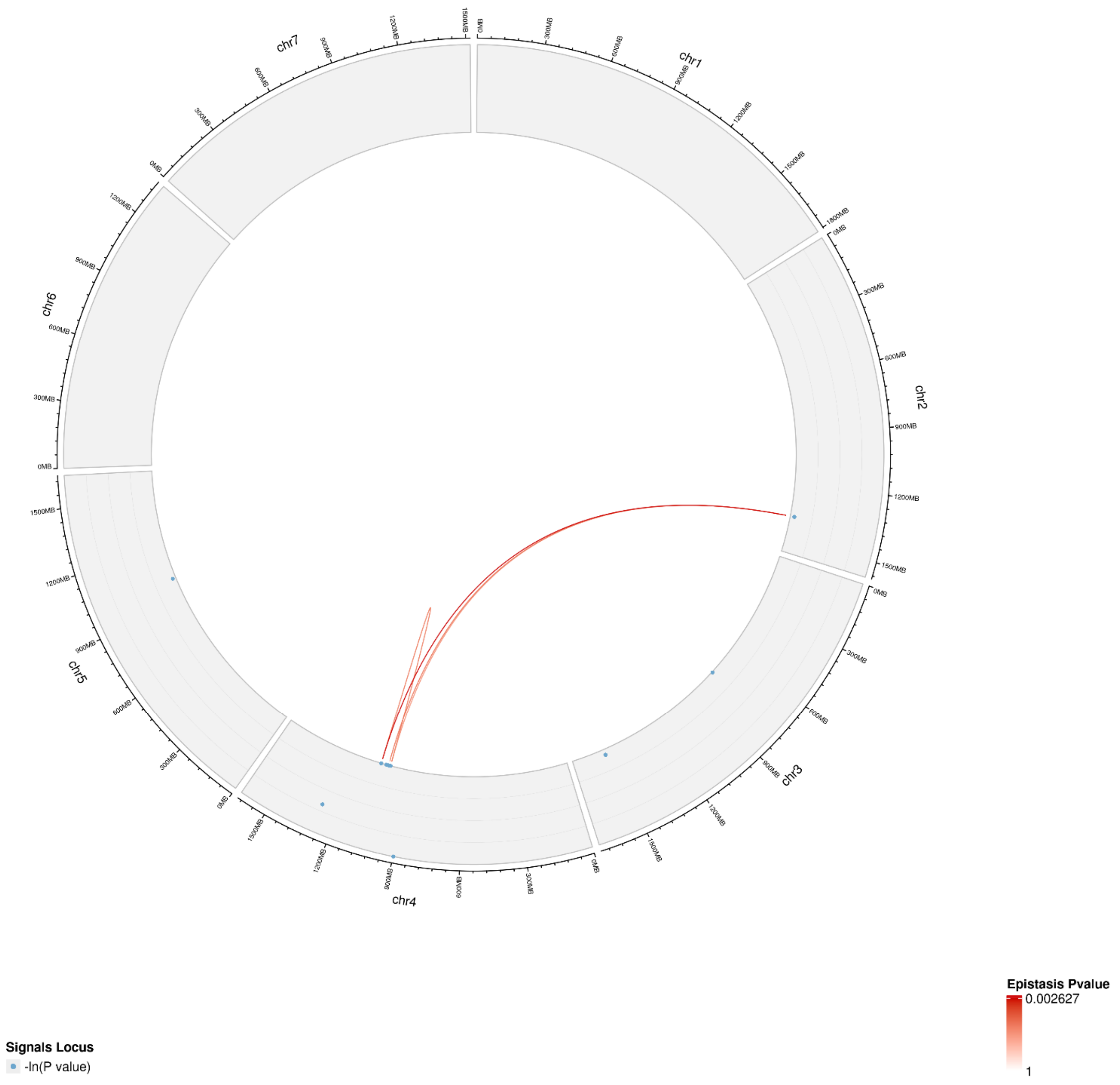

Supplementary Figure S2. Epistatic Interactions Between SNP Pairs on Chromosomes 2 and 4

1.2 Supplementary Tables

Supplementary Table S1. Epistatic Interactions Between SNP Pairs on Chromosomes 2 and 4

CHR1	SNP1	CHR2	SNP2	OR_INT	STAT	P	R^2_percent(%)	FDR_adjusted_P
chr2	chr2::1370936770	chr4	chr4::1013887633	0.0381873	3.85698	0.04954	1.9731	0.049540
chr2	chr2::1370936770	chr4	chr4::1025369220	6.30227	5.27697	0.02161	2.301	0.034853
chr2	chr2::1370936770	chr4	chr4::1068677594	0.00612837	9.05007	0.002627	2.7145	0.010508
chr4	chr4::1013887633	chr4	chr4::1068677594	533.852	4.94701	0.02614	2.0314	0.034853

CHR1: Chromosome 1

SNP1: SNP identifier 1

CHR2: Chromosome 2

SNP2: SNP identifier 2

BETA_INT/OR_INT: Coefficient from linear/logistic regression

STAT: Test statistic from linear/logistic regression

P: Significance p-value from linear/logistic regression

R^2_percent (%): Percentage of phenotypic variation explained by epistasis

FDR_adjusted_P: P-value adjusted for multiple testing using the False Discovery Rate (FDR) method

Supplementary Table S2. F_2 individuals selected for GWAS analysis

Sample ID	Wing petal type	Crossing pair
FTS0007	Short wing petal	‘Yundoulvxin 1’ (short winged) × ‘Yundou 1183’
FTS0014	Normal wing petal	‘Yundoulvxin 1’ (short winged) × ‘Yundou 1183’
FTS0027	Normal wing petal	‘Yundoulvxin 1’ (short winged) × ‘Yundou 1183’
FTS0054	Normal wing petal	‘Yundoulvxin 1’ (short winged) × ‘Yundou 1183’
FTS0063	Normal wing petal	‘Yundoulvxin 1’ (short winged) × ‘Yundou 1183’
FTS0092	Normal wing petal	‘Yundoulvxin 1’ (short winged) × ‘Yundou 1183’
FTS0093	Normal wing petal	‘Yundoulvxin 1’ (short winged) × ‘Yundou 1183’
FTS0094	Normal wing petal	‘Yundoulvxin 1’ (short winged) × ‘Yundou 1183’
FTS0105	Normal wing petal	‘Yundoulvxin 1’ (short winged) × ‘Yundou 1183’
FTS0106	Normal wing petal	‘Yundoulvxin 1’ (short winged) × ‘Yundou 1183’
FTS0170	Normal wing petal	‘Yundoulvxin 1’ (short winged) × ‘Yundou 1183’
FTS0211	Normal wing petal	‘Yundoulvxin 1’ (short winged) × ‘Yundou 1183’
FTS0213	Short wing petal	‘Yundoulvxin 1’ (short winged) × ‘Yundou 1183’
FTS0215	Normal wing petal	‘Yundoulvxin 1’ (short winged) × ‘Yundou 1183’

FTS0220	Normal wing petal	‘Yundoulvxin 1’ (short winged) × ‘Yundou 1183’
FTS0225	Normal wing petal	‘Yundoulvxin 1’ (short winged) × ‘Yundou 1183’
FTS0241	Normal wing petal	‘Yundoulvxin 1’ (short winged) × ‘Yundou 1183’
FTS0277	Normal wing petal	‘Yundoulvxin 1’ (short winged) × ‘Yundou 1183’
FTS0307	Normal wing petal	‘Yundoulvxin 1’ (short winged) × ‘Yundou 1183’
FTS0309	Normal wing petal	‘Yundoulvxin 1’ (short winged) × ‘Yundou 1183’
FTS0343	Normal wing petal	‘Yundoulvxin 1’ (short winged) × ‘Yundou 1183’
FTS0346	Short wing petal	‘Yundoulvxin 1’ (short winged) × ‘Yundou 1183’
FTS0357	Normal wing petal	‘Yundoulvxin 1’ (short winged) × ‘Yundou 1183’
FTS0380	Normal wing petal	‘Yundoulvxin 1’ (short winged) × ‘Yundou 1183’
FTS0390	Normal wing petal	‘Yundoulvxin 1’ (short winged) × ‘Yundou 1183’
FTS0395	Normal wing petal	‘Yundoulvxin 1’ (short winged) × ‘Yundou 1183’
FTS0435	Normal wing petal	‘Yundoulvxin 1’ (short winged) × ‘Yundou 1183’
FTS0465	Short wing petal	‘Yundoulvxin 1’ (short winged) × ‘Yundou 1183’
FTS0467	Short wing petal	‘Yundoulvxin 1’ (short winged) × ‘Yundou 1183’

FTS0473	Normal wing petal	'Yundoulvxin 1' (short winged) × 'Yundou 1183'
FTS0482	Normal wing petal	'Yundoulvxin 1' (short winged) × 'Yundou 1183'
FTS0483	Short wing petal	'Yundoulvxin 1' (short winged) × 'Yundou 1183'
FTS0510	Short wing petal	'Yundoulvxin 1' (short winged) × 'Yundou 1183'
FTS0511	Normal wing petal	'Yundoulvxin 1' (short winged) × 'Yundou 1183'
FTS0536	Normal wing petal	'Yundoulvxin 1' (short winged) × 'Yundou 1183'
FTS0570	Normal wing petal	'Yundoulvxin 1' (short winged) × 'Yundou 1183'
FTS0571	Normal wing petal	'Yundoulvxin 1' (short winged) × 'Yundou 1183'
FTS0572	Normal wing petal	'Yundoulvxin 1' (short winged) × 'Yundou 1183'
FTS0588	Normal wing petal	'Yundoulvxin 1' (short winged) × 'Yundou 1183'
FTS0604	Normal wing petal	'Yundoulvxin 1' (short winged) × 'Yundou 1183'
FTS0619	Normal wing petal	'Yundoulvxin 1' (short winged) × 'Yundou 1183'
FTS0671	Normal wing petal	'Yundoulvxin 1' (short winged) × 'Yundou 1183'
FTS0674	Normal wing petal	'Yundoulvxin 1' (short winged) × 'Yundou 1183'
FTS0678	Normal wing petal	'Yundoulvxin 1' (short winged) × 'Yundou 1183'

FTS0693	Normal wing petal	‘Yundoulvxin 1’ (short winged) × ‘Yundou 1183’
FTS0731	Normal wing petal	‘Yundoulvxin 1’ (short winged) × ‘Yundou 1183’
FTS0732	Normal wing petal	‘Yundoulvxin 1’ (short winged) × ‘Yundou 1183’
FTS0735	Normal wing petal	‘Yundoulvxin 1’ (short winged) × ‘Yundou 1183’
FTS0744	Normal wing petal	‘Yundoulvxin 1’ (short winged) × ‘Yundou 1183’
FTS0750	Normal wing petal	‘Yundoulvxin 1’ (short winged) × ‘Yundou 1183’
FTS0763	Normal wing petal	‘Yundoulvxin 1’ (short winged) × ‘Yundou 1183’
FTS0788	Normal wing petal	‘Yundoulvxin 1’ (short winged) × ‘Yundou 1183’
FTS0789	Short wing petal	‘Yundoulvxin 1’ (short winged) × ‘Yundou 1183’
FTS0790	Short wing petal	‘Yundoulvxin 1’ (short winged) × ‘Yundou 1183’
FTS0822	Short wing petal	‘Yundoulvxin 1’ (short winged) × ‘Yundou 1183’
FTS0824	Normal wing petal	‘Yundoulvxin 1’ (short winged) × ‘Yundou 1183’
FTS0831	Short wing petal	‘Yundoulvxin 1’ (short winged) × ‘Yundou 1183’
FTS0850	Normal wing petal	‘Yundoulvxin 1’ (short winged) × ‘Yundou 1183’
FTS0857	Normal wing petal	‘Yundoulvxin 1’ (short winged) × ‘Yundou 1183’

FTS0863	Normal wing petal	‘Yundoulvxin 1’ (short winged) × ‘Yundou 1183’
FTS0889	Normal wing petal	‘Yundoulvxin 1’ (short winged) × ‘Yundou 1183’
FTS0906	Normal wing petal	‘Yundoulvxin 1’ (short winged) × ‘Yundou 1183’
FTS0988	Normal wing petal	‘Yundoulvxin 1’ (short winged) × ‘Yundou 1183’
FTS1013	Short wing petal	‘Yundoulvxin 1’ (short winged) × ‘Yundou 1183’
FTS1015	Normal wing petal	‘Yundoulvxin 1’ (short winged) × ‘Yundou 1183’
FTS1016	Normal wing petal	‘Yundoulvxin 1’ (short winged) × ‘Yundou 1183’
FTS1020	Short wing petal	‘Yundoulvxin 1’ (short winged) × ‘Yundou 1183’
FTS1021	Normal wing petal	‘Yundoulvxin 1’ (short winged) × ‘Yundou 1183’
FTS1023	Normal wing petal	‘Yundoulvxin 1’ (short winged) × ‘Yundou 1183’
FTS1104	Normal wing petal	‘Yundoulvxin 1’ (short winged) × ‘Yundou 1183’
FTS1191	Normal wing petal	‘Yundoulvxin 1’ (short winged) × ‘Yundou 1183’
FTS1224	Normal wing petal	‘Yundoulvxin 1’ (short winged) × ‘Yundou 1183’
FTS1256	Normal wing petal	‘Yundoulvxin 1’ (short winged) × ‘Yundou 1183’
FTS1274	Normal wing petal	‘Yundoulvxin 1’ (short winged) × ‘Yundou 1183’

FTS1328	Normal wing petal	‘Yundoulvxin 1’ (short winged) × ‘Yundou 1183’
FTS1373	Normal wing petal	‘Yundoulvxin 1’ (short winged) × ‘Yundou 1183’
FTS1407	Normal wing petal	‘Yundoulvxin 1’ (short winged) × ‘Yundou 1183’
FTS1433	Short wing petal	‘Yundoulvxin 1’ (short winged) × ‘Yundou 1183’
FTS1434	Short wing petal	‘Yundoulvxin 1’ (short winged) × ‘Yundou 1183’
FTS1440	Normal wing petal	‘Yundoulvxin 1’ (short winged) × ‘Yundou 1183’
FTS1448	Short wing petal	‘Yundoulvxin 1’ (short winged) × ‘Yundou 1183’
FTS1451	Normal wing petal	‘Yundoulvxin 1’ (short winged) × ‘Yundou 1183’
FTS1477	Short wing petal	‘Yundoulvxin 1’ (short winged) × ‘Yundou 1183’
FTS1513	Short wing petal	‘Yundoulvxin 1’ (short winged) × ‘Yundou 1183’
FTS1517	Short wing petal	‘Yundoulvxin 1’ (short winged) × ‘Yundou 1183’
FTS1538	Normal wing petal	‘Yundoulvxin 1’ (short winged) × ‘Yundou 1183’
FTS1539	Short wing petal	‘Yundoulvxin 1’ (short winged) × ‘Yundou 1183’
FTS1545	Normal wing petal	‘Yundoulvxin 1’ (short winged) × ‘Yundou 1183’
FTS1569	Normal wing petal	‘Yundoulvxin 1’ (short winged) × ‘Yundou 1183’

FTS1603	Normal wing petal	‘Yundoulvxin 1’ (short winged) × ‘Yundou 1183’
FTS1609	Normal wing petal	‘Yundoulvxin 1’ (short winged) × ‘Yundou 1183’
FTS1628	Normal wing petal	‘Yundoulvxin 1’ (short winged) × ‘Yundou 1183’
FTS1643	Normal wing petal	‘Yundoulvxin 1’ (short winged) × ‘Yundou 1183’
FTS1645	Short wing petal	‘Yundoulvxin 1’ (short winged) × ‘Yundou 1183’
FTS1648	Short wing petal	‘Yundoulvxin 1’ (short winged) × ‘Yundou 1183’
FTS1653	Normal wing petal	‘Yundoulvxin 1’ (short winged) × ‘Yundou 1183’
FTS1654	Normal wing petal	‘Yundoulvxin 1’ (short winged) × ‘Yundou 1183’
FTS1666	Normal wing petal	‘Yundoulvxin 1’ (short winged) × ‘Yundou 1183’
FTS1689	Normal wing petal	‘Yundoulvxin 1’ (short winged) × ‘Yundou 1183’
FTS1700	Short wing petal	‘Yundoulvxin 1’ (short winged) × ‘Yundou 1183’
FTS1787	Normal wing petal	‘Yundoulvxin 1’ (short winged) × ‘Yundou 1183’
FTS1808	Normal wing petal	‘Yundoulvxin 1’ (short winged) × ‘Yundou 1183’
FTS1975	Normal wing petal	‘K0692’ (short winged) × ‘Yundou 1183’
FTS1990	Normal wing petal	‘K0692’ (short winged) × ‘Yundou 1183’
FTS1993	Normal wing petal	‘K0692’ (short winged) × ‘Yundou 1183’
FTS1994	Short wing petal	‘K0692’ (short winged) × ‘Yundou 1183’

FTS2009	Normal wing petal	‘K0692’ (short winged) × ‘Yundou 1183’
FTS2012	Normal wing petal	‘K0692’ (short winged) × ‘Yundou 1183’
FTS2057	Short wing petal	‘K0692’ (short winged) × ‘Yundou 1183’
FTS2061	Normal wing petal	‘K0692’ (short winged) × ‘Yundou 1183’
FTS2085	Short wing petal	‘K0692’ (short winged) × ‘Yundou 1183’
FTS2090	Normal wing petal	‘K0692’ (short winged) × ‘Yundou 1183’
FTS2133	Short wing petal	‘K0692’ (short winged) × ‘Yundou 1183’
FTS2134	Normal wing petal	‘K0692’ (short winged) × ‘Yundou 1183’
FTS2140	Normal wing petal	‘K0692’ (short winged) × ‘Yundou 1183’
FTS2174	Normal wing petal	‘K0692’ (short winged) × ‘Yundou 1183’
FTS2175	Short wing petal	‘K0692’ (short winged) × ‘Yundou 1183’
FTS2224	Normal wing petal	‘K0692’ (short winged) × ‘Yundou 1183’
FTS2253	Normal wing petal	‘K0692’ (short winged) × ‘Yundou 1183’
FTS2287	Normal wing petal	‘K0692’ (short winged) × ‘Yundou 1183’
FTS2300	Normal wing petal	‘K0692’ (short winged) × ‘Yundou 1183’
FTS2307	Short wing petal	‘K0692’ (short winged) × ‘Yundou 1183’
FTS2337	Short wing petal	‘K0692’ (short winged) × ‘Yundou 1183’
FTS2376	Short wing petal	‘K0692’ (short winged) × ‘Yundou 1183’
FTS2380	Normal wing petal	‘K0692’ (short winged) × ‘Yundou 1183’
FTS2422	Normal wing petal	‘K0692’ (short winged) × ‘Yundou 1183’
FTS2429	Normal wing petal	‘K0692’ (short winged) × ‘Yundou 1183’
FTS2509	Normal wing petal	‘K0692’ (short winged) × ‘Yundou 1183’
FTS2534	Normal wing petal	‘K0692’ (short winged) × ‘Yundou 1183’
FTS2540	Normal wing petal	‘K0692’ (short winged) × ‘Yundou 1183’
FTS2648	Short wing petal	‘K0692’ (short winged) × ‘Yundou 1183’

FTS2676	Short wing petal	‘K0692’ (short winged) × ‘Yundou 1183’
FTS2699	Normal wing petal	‘K0692’ (short winged) × ‘Yundou 1183’
FTS2719	Short wing petal	‘K0692’ (short winged) × ‘Yundou 1183’
FTS2781	Normal wing petal	‘K0692’ (short winged) × ‘Yundou 1183’
FTS2785	Normal wing petal	‘K0692’ (short winged) × ‘Yundou 1183’
FTS2788	Short wing petal	‘K0692’ (short winged) × ‘Yundou 1183’
FTS2794	Normal wing petal	‘K0692’ (short winged) × ‘Yundou 1183’
FTS2805	Normal wing petal	‘K0692’ (short winged) × ‘Yundou 1183’
FTS2811	Short wing petal	‘K0692’ (short winged) × ‘Yundou 1183’
FTS2812	Normal wing petal	‘K0692’ (short winged) × ‘Yundou 1183’
FTS2818	Normal wing petal	‘K0692’ (short winged) × ‘Yundou 1183’
FTS2823	Short wing petal	‘K0692’ (short winged) × ‘Yundou 1183’
FTS2832	Normal wing petal	‘K0692’ (short winged) × ‘Yundou 1183’
FTS2836	Normal wing petal	‘K0692’ (short winged) × ‘Yundou 1183’
FTS2847	Normal wing petal	‘K0692’ (short winged) × ‘Yundou 1183’
FTS2861	Short wing petal	‘K0692’ (short winged) × ‘Yundou 1183’
FTS2871	Short wing petal	‘K0692’ (short winged) × ‘Yundou 1183’
FTS2881	Normal wing petal	‘K0692’ (short winged) × ‘Yundou 1183’
FTS2934	Short wing petal	‘K0692’ (short winged) × ‘Yundou 1183’
FTS3030	Short wing petal	‘K0692’ (short winged) × ‘Yundou 1183’
FTS3043	Normal wing petal	‘K0692’ (short winged) × ‘Yundou 1183’
FTS3065	Normal wing petal	‘K0692’ (short winged) × ‘Yundou 1183’
FTS3073	Normal wing petal	‘K0692’ (short winged) × ‘Yundou 1183’
FTS3104	Normal wing petal	‘K0692’ (short winged) × ‘Yundou 1183’
FTS3117	Normal wing petal	‘K0692’ (short winged) × ‘Yundou 1183’

FTS3129	Normal wing petal	‘K0692’ (short winged) × ‘Yundou 1183’
FTS3149	Normal wing petal	‘K0692’ (short winged) × ‘Yundou 1183’
FTS3156	Normal wing petal	‘K0692’ (short winged) × ‘Yundou 1183’
FTS3188	Normal wing petal	‘K0692’ (short winged) × ‘Yundou 1183’
FTS3204	Short wing petal	‘K0692’ (short winged) × ‘Yundou 1183’
FTS3245	Normal wing petal	‘K0692’ (short winged) × ‘Yundou 1183’
FTS3256	Normal wing petal	‘K0692’ (short winged) × ‘Yundou 1183’
FTS3291	Normal wing petal	‘K0692’ (short winged) × ‘Yundou 1183’
FTS3326	Normal wing petal	‘K0692’ (short winged) × ‘Yundou 1183’
FTS3429	Normal wing petal	‘K0692’ (short winged) × ‘Yundou 1183’
FTS3432	Normal wing petal	‘K0692’ (short winged) × ‘Yundou 1183’
FTS3434	Normal wing petal	‘K0692’ (short winged) × ‘Yundou 1183’
FTS3503	Normal wing petal	‘K0692’ (short winged) × ‘Yundou 1183’
FTS3514	Short wing petal	‘K0692’ (short winged) × ‘Yundou 1183’
FTS3544	Normal wing petal	‘K0692’ (short winged) × ‘Yundou 1183’
FTS3571	Normal wing petal	‘K0692’ (short winged) × ‘Yundou 1183’
FTS3590	Normal wing petal	‘K0692’ (short winged) × ‘Yundou 1183’
FTS3602	Short wing petal	‘K0692’ (short winged) × ‘Yundou 1183’
FTS3610	Normal wing petal	‘K0692’ (short winged) × ‘Yundou 1183’
FTS3649	Normal wing petal	‘K0692’ (short winged) × ‘Yundou 1183’
FTS3688	Normal wing petal	‘K0692’ (short winged) × ‘Yundou 1183’
FTS3734	Normal wing petal	‘K0692’ (short winged) × ‘Yundou 1183’
FTS3747	Normal wing petal	‘K0692’ (short winged) × ‘Yundou 1183’
FTS3757	Short wing petal	‘K0692’ (short winged) × ‘Yundou 1183’
FTS3785	Short wing petal	‘K0692’ (short winged) × ‘Yundou 1183’

FTS3815	Normal wing petal	‘K0692’ (short winged) × ‘Yundou 1183’
FTS3834	Short wing petal	‘K0692’ (short winged) × ‘Yundou 1183’
FTS3845	Normal wing petal	‘K0692’ (short winged) × ‘Yundou 1183’
FTS3918	Normal wing petal	‘K0692’ (short winged) × ‘Yundou 1183’
FTS3955	Normal wing petal	‘K0692’ (short winged) × ‘Yundou 1183’
FTS3961	Normal wing petal	‘K0692’ (short winged) × ‘Yundou 1183’
FTS3963	Normal wing petal	‘K0692’ (short winged) × ‘Yundou 1183’
FTS3967	Normal wing petal	‘K0692’ (short winged) × ‘Yundou 1183’
FTS3971	Normal wing petal	‘K0692’ (short winged) × ‘Yundou 1183’
FTS4006	Normal wing petal	‘K0692’ (short winged) × ‘Yundou 1183’
FTS4007	Normal wing petal	‘K0692’ (short winged) × ‘Yundou 1183’
FTS4039	Normal wing petal	‘K0692’ (short winged) × ‘Yundou 1183’
FTS4086	Normal wing petal	‘K0692’ (short winged) × ‘Yundou 1183’
FTS4096	Normal wing petal	‘K0692’ (short winged) × ‘Yundou 1183’
FTS4104	Short wing petal	‘K0692’ (short winged) × ‘Yundou 1183’
FTS4110	Normal wing petal	‘K0692’ (short winged) × ‘Yundou 1183’
FTS4125	Normal wing petal	‘K0692’ (short winged) × ‘Yundou 1183’
FTS4170	Short wing petal	‘K0692’ (short winged) × ‘Yundou 1183’
FTS4173	Short wing petal	‘K0692’ (short winged) × ‘Yundou 1183’
